# Colliding public health priorities: A call to improve the understanding of autistic individuals utilizing housing assistance

**DOI:** 10.1371/journal.pone.0315008

**Published:** 2024-12-20

**Authors:** Lindsay Shea, Anne Roux, Amy Blank Wilson, Jonas Ventimiglia, Conner Carlton, Wei-Lin Lee, Dylan Cooper, Shelby Frisbie

**Affiliations:** 1 A.J. Drexel Autism Institute, Drexel University, Philadelphia, PA, United States of America; 2 School of Social Work, University of North Carolina–Chapel Hill, Chapel Hill, NC, United States of America; Wingate University, UNITED STATES OF AMERICA

## Abstract

The objective of this study was to identify utilization of housing support provided by the U.S. Department of Housing and Urban Development (HUD) among autistic people in the U.S. Using 2008 and 2016 Medicaid data, we identified autistic individuals birth to 61 years and linked them to national HUD data. We characterized demographics, co-occurring conditions, and HUD program involvement. Autistic Medicaid enrollees enrolled in HUD increased by 70% between 2008 and 2016. Among 846,350 autistic Medicaid enrollees in 2016, 10.4% (n = 88,315) were HUD-assisted. HUD-assisted autistic individuals, versus non-HUD-assisted, were more likely to be Black/African American and less likely to have private insurance. Most lived in urban areas and were enrolled in the Housing Choice Voucher program. Approximately 2,600 autistic individuals (3%) were homeless at HUD entry. Growing numbers of HUD-assisted autistic individuals point toward an urgent need for federal data to understand and address public health contexts of housing affordability and instability to complement existing clinical autism research investments. Integrated public health, housing, and disability supports must address equitable income supports and housing assistance needed to support the health needs of autistic individuals.

## Introduction

Increasing prevalence of autism, the fastest growing developmental disability in the U.S. [1], and a national housing crisis, represent distinct, multifaceted public health priorities that are typically examined separately. Datasets, generated from the multiple, siloed health, housing, and disability service delivery systems which autistic people and their families to navigate, have rarely been linked to examine these cross-system experiences. To date, autism research has mainly focused on the clinical aspects of autism diagnosis, primarily among children and, largely lacks a public health approach [2].

Affordable, quality housing fundamentally shapes health outcomes [3, 4]. Despite people with disabilities representing a significant share of the population in need of housing assistance [5], and the potential for compounding health risks while living under these circumstances [5, 6], the intersection of housing and health among autistic people is largely unknown. The autistic population experiences increased rates of suboptimal health and mental health outcomes, low employment rates, and poor self-reported wellbeing even in stable housing situations [7, 8]. When research has included autistic individuals from minoritized communities (largely focused on children) [9], adverse health and mental health outcomes observed are more frequently observed and reported [10–13]. Unaffordable housing may exacerbate these disparities and impede access to needed services within this population across the lifespan, given that housing insecurity generates financial strain limiting ability to navigate services.

In the U.S., Medicaid is the primary source of health insurance for autistic people. Medicaid covers community-based services but does not typically cover housing costs. The Department of Housing and Urban Development (HUD) is the primary federal source of housing assistance in the U.S, available to people with developmental disabilities like autism. Eligibility for HUD support is determined based on annual gross income, disability or elderly status (or a family member of a person in these groups), family size, and US citizenship.

HUD administers three predominant programs: multifamily housing (MF) which subsidizes rent in multi-unit properties, public housing (PH) which provides affordable rental housing units, and the Housing Choice Voucher (HCV) program which is the largest source of rental assistance in the nation for tenants [6]. The innovative use of linked Medicaid and HUD data can explicate the relationship between housing assistance and health among autistic people to inform comprehensive public health strategies to address the care needs of this growing group. This study is the first known examination of linked HUD and Medicaid data to characterize the population of autistic people receiving HUD assistance.

The [BLINDED FOR PEER REVIEW] IRB approved this study, including a waiver of informed consent, for the use of national claims data. Data for this study were accessed from January 1, 2024 through August 1, 2024. No contact information for individual participants was accessed.

## Materials and methods

Autistic individuals were identified from the national Medicaid Analytics eXtract files (MAX) and the T-MSIS Analytic Files (TAF) using claims from January 1 to December 31 in 2008 and 2016. Beneficiary IDs were deduplicated across data years. We identified an autism diagnosis using a validated claims-based algorithm generated by the Chronic Conditions Warehouse (CCW) developed and operated by the Centers for Medicare and Medicaid Services (CMS) that requires either two outpatient claims or one inpatient diagnosis code (ICD-9 299.x or ICD-10 F84.X) on a claim during the study period [14]. To address administrative churning, we included individuals with at least 9 months of Medicaid enrollment during a consecutive 12-month period during the study period.

We created Medicaid-HUD linked datasets for 2008 and 2016 using social security numbers and limited to individuals 0–61 years of age, the definition of “non-elderly” in HUD data, and tracked the number of autistic HUD-assisted individuals each year. We examined HUD program type, geographic region, urbanicity, duration of enrollment, number of people in the household, homelessness at HUD entry, and median family income for 2016. HUD program types included Public Housing, Multi-Family Housing, and Housing Choice Vouchers.

We characterized the cohort of autistic individuals receiving HUD assistance by age group, sex, race/ethnicity, and health insurance. Co-occurring intellectual disability was also identified using a CCW validated algorithm [14]. Analyses were performed using SAS version 9.4. [BLINDED] University’s Institutional Review Board approved this study, including a waiver of informed consent.

## Results

There were 52,924 autistic Medicaid enrollees represented in the HUD data in 2008 (Table 1). This increased by 70% to 88,315 individuals in 2016. Thus, 10.4% of the 846,350 autistic Medicaid enrollees in 2016 were HUD-assisted. Among this group, 10,334 autistic individuals lived with at least one other autistic person. During the same period, the overall population of HUD-assisted individuals decreased slightly from 11,608,614 to 11,507,157. A greater proportion of HUD-assisted autistic individuals were Black/African American than their counterparts without HUD enrollment (33.3% versus 13.5%). HUD-assisted autistic individuals were less likely to have private insurance, and 72.2% were children under age 18.

**Table 1:** Sample characteristics of autistic Medicaid enrollees in 2016.

	HUD Involvement		No HUD Involvement	
	(N = 88,315)		(N = 846,350)	
	N	%	N	%
**Age**				
0–17	63,782	72.22%	590,785	69.80%
18–25	11,130	12.60%	138,074	16.31%
26–45	9,738	11.03%	91,033	10.76%
46–61	3,665	4.15%	26,458	3.13%
**Race/Ethnicity**				
Asian/Pacific Islander	1,304	1.48%	25,002	2.95%
Black/African American	29,376	33.26%	114,240	13.50%
Hispanic/Latino	14,934	16.91%	150,245	17.75%
Native Alaskan/American	578	0.65%	7,065	0.83%
White	32,805	37.15%	447,801	52.91%
Multi-race	1,349	1.53%	9,632	1.14%
Missing	7,969	9.02%	92,365	10.91%
**Female**	21,216	24.02%	194,871	23.02%
**Insurance (in addition to Medicaid)**				
Private	8,320	9.42%	166,116	19.63%
Medicare	10,616	12.02%	94,376	11.15%
**Co-occurring ID**	21,868	24.76%	229,566	27.12%
**Hud Program Type** ^**a**^				
HCV	49,270	58.84%		
PH	17,777	21.23%		
MF	16,633	0.1986		
Other (none of the above)	52	0.06%		
**Region**				
Northeast	24,999	29.86%		
Midwest	18,596	22.21%		
South	22,156	26.46%		
West	13,350	15.94%		
U.S. Territories	4,631	5.53%		
**Urbanicity**				
Urban	62,783	74.98%		
Suburban	12,785	15.27%		
Rural	1,503	1.80%		
Missing	6,661	7.96%		
**Duration in HUD (mean)**	6.74 years			
**# in household**				
1	11,906	14.22%		
2	17,472	20.87%		
3	21,042	25.13%		
4	16,507	19.71%		
5+	16,805	20.07%		
**Homeless at entry**	2,669	3.02%		
**Income** ^**b**^				
Total annual income	$16,754			
Very low income	76,560	91.43%		
Extremely low income	54,765	65.41%		

Notes:

^a^HUD types: HCV = Housing Choice Vouchers, PH = Public Housing, MF = Multi-family Housing.

^b^Income is measured as the median family income. Very Low Income = families with incomes not exceeding 50% of the median family income for their area (or the federal poverty guidelines), Extremely Low Income = families with incomes not exceeding 30% of the medican family income for the area. Source of income information: https://www.huduser.gov/portal/datasets/il//il24/IncomeLimitsMethodology-FY24.pdf

Nearly 60% of autistic HUD-assisted individuals received the Housing Choice Voucher, and 21% were enrolled in Public Housing. The mean duration of HUD assistance was 6.7 years, and 20% of autistic HUD-assisted individuals resided in households with five or more individuals. Most 75% lived in urban areas, and in northeast or south regions of the U.S. A small proportion (3%) were homeless at HUD entry, although this totaled over 2,600 autistic individuals (82% under the age of 18). The median family income for HUD-assisted households with an autistic individual was $16,754, and 65% met HUD’s criteria for extremely low income (i.e. 30% of the Area Median Income or less).

## Discussion

Examining the intersection of public health priorities, including housing assistance and autism prevalence, is increasingly urgent as the escalating housing crisis severely impacts disabled people. Increasing numbers of HUD-assisted autistic individuals, and the frequency of extremely low-income families within this group, underscores growing financial hardship within this population. These individuals likely need comprehensive support systems that integrate financial assistance with housing, healthcare, transportation, and employment [15]. Nearly three-fourths of HUD-assisted autistic Medicaid enrollees were children, compared to a rate of 35% in the overall HUD population [16]. Lower enrollment among autistic adults may reflect lack of capacity to identify and serve autistic adults as noted in other public service systems [17].

Our findings elucidate a need for preemptive interventions to identify autistic individuals at risk for homelessness, including a need for new and modified assessment and service planning tools, and to provide stabilization resources, including emergency housing options. A recent survey of Georgians with developmental disabilities found that 4.2% were experiencing homelessness–more than twice the rate of homelessness among non-disabled Georgia residents [18].

Disproportionately higher rates of HUD enrollment among Black/African American autistic individuals echo concerns that minoritized populations face compounded disadvantages, including increased exposure to environmental and social stressors and disproportionate barriers to service access, that exacerbate health disparities [19]. A larger share of HUD-assisted autistic individuals resided in urban areas which feature unique barriers regarding transportation, crime, healthcare access, and food deserts. Continued observations of housing and service access among rural autistic populations are needed to assess geographically equitable public health strategies.

The predominance of autistic individuals in the Housing Choice Voucher program suggests that this program may be particularly beneficial due to its opportunities for autonomy and community integration but suggests a need for support to navigate options in a complex housing market. Very few autistic individuals (1,979 or 2.24% of autistic HUD-supported individuals) used Multifamily Section 811 Supportive Housing for Persons with Disabilities, indicating that the reach of this disability-focused program to autistic individuals may be limited. Across programs, autistic individuals relied on HUD support for extended periods (mean of 6.7 years), emphasizing the need for sustained and stable housing options that can adapt to the long-term care needs of autistic individuals.

Limitations of this research include reliance upon linked claims data collected for billing purposes. Claims did not represent autistic HUD-assisted people who were not enrolled in Medicaid. The extensive timeline to acquire and link data precluded analysis of more recent data, although current findings provide a novel contribution and establish a baseline for future comparisons.

Intertwined challenges of increasing autism prevalence and housing gaps must be examined together, given their additive effects. Federal action to catalyze timely data access is key for building the public health evidence base and prioritizing opportunities to advance access to affordable and stable housing among people with disabilities. Linked HUD-Medicaid data propels research that reflects real-world experiences across populations. Populations with rapidly increasing prevalence, including autism, have an inherently reduced evidence base which hinders the prediction, definition, and calibration of programs to meet their needs. Findings emphasize the importance of cataloging the impact of systemic racism and disparities across systems. Future research should explore outcomes of housing assistance among autistic individuals to identify key policy strategies (e.g. emerging Medicaid waivers that include housing supports and the waiting list for HUD programs).
