# Supplementary material for: Colliding public health priorities: A call to improve the understanding of autistic individuals utilizing housing assistance
Source: PLoS One. 2024 Dec 20;19(12):e0315008. doi: 10.1371/journal.pone.0315008 (PMC11661572; doi:10.1371/journal.pone.0315008)
Supplement: Peer review history

# Peer review: Decision Letter 0

**DOI:** 10.1371/journal.pone.0315008.r001

**Authors:** Edward Chiyaka

4 Oct 2024

PONE-D-24-33807Colliding Public Health Priorities: A Call to Improve the Understanding of Autistic Individuals Utilizing Housing AssistancePLOS ONE

Dear Dr. Shea,

Thank you for submitting your manuscript to PLOS ONE. After careful consideration, we feel that it has merit but does not fully meet PLOS ONE’s publication criteria as it currently stands. Therefore, we invite you to submit a revised version of the manuscript that addresses the points raised during the review process.

==============================

**In addition to addressing the reviewers, please also consider the following.** 

It seems that your current keywords, such as "disability" and "policy," do not align with the content of your article, as these terms are not mentioned anywhere in the text. Remember, your keywords should accurately reflect the central themes of your research and make your article more discoverable online. Consider revising your keywords to better describe the scope and findings of your study, ensuring they help people find your work when conducting relevant searches.

==============================

Please submit your revised manuscript by Nov 18 2024 11:59PM. If you will need more time than this to complete your revisions, please reply to this message or contact the journal office at plosone@plos.org. When you're ready to submit your revision, log on to https://www.editorialmanager.com/pone/ and select the 'Submissions Needing Revision' folder to locate your manuscript file.

Please include the following items when submitting your revised manuscript:

- A rebuttal letter that responds to each point raised by the academic editor and reviewer(s). You should upload this letter as a separate file labeled 'Response to Reviewers'.
- A marked-up copy of your manuscript that highlights changes made to the original version. You should upload this as a separate file labeled 'Revised Manuscript with Track Changes'.
- An unmarked version of your revised paper without tracked changes. You should upload this as a separate file labeled 'Manuscript'.

If you would like to make changes to your financial disclosure, please include your updated statement in your cover letter. Guidelines for resubmitting your figure files are available below the reviewer comments at the end of this letter.

If applicable, we recommend that you deposit your laboratory protocols in protocols.io to enhance the reproducibility of your results. Protocols.io assigns your protocol its own identifier (DOI) so that it can be cited independently in the future. For instructions see: https://journals.plos.org/plosone/s/submission-guidelines#loc-laboratory-protocols. Additionally, PLOS ONE offers an option for publishing peer-reviewed Lab Protocol articles, which describe protocols hosted on protocols.io. Read more information on sharing protocols at https://plos.org/protocols?utm_medium=editorial-email&utm_source=authorletters&utm_campaign=protocols.

We look forward to receiving your revised manuscript.

Kind regards,

Edward Chiyaka, Ph.D., MSc

Academic Editor

PLOS ONE

Journal Requirements: When submitting your revision, we need you to address these additional requirements. 1. Please ensure that your manuscript meets PLOS ONE's style requirements, including those for file naming. The PLOS ONE style templates can be found at https://journals.plos.org/plosone/s/file?id=wjVg/PLOSOne_formatting_sample_main_body.pdf and https://journals.plos.org/plosone/s/file?id=ba62/PLOSOne_formatting_sample_title_authors_affiliations.pdf 2. Thank you for stating the following financial disclosure: "This project is supported by the Health Resources and Services Administration (HRSA) of the U.S. Department of Health and Human Services (HHS) under cooperative agreement UT6MC45902 Autism Transitions Research Project. The information, content, and/or conclusions are those of the author and should not be construed as the official position or policy of, nor should any endorsements be inferred by HRSA, HHS or the U.S. Government." Please state what role the funders took in the study.  If the funders had no role, please state: ""The funders had no role in study design, data collection and analysis, decision to publish, or preparation of the manuscript."" If this statement is not correct you must amend it as needed. Please include this amended Role of Funder statement in your cover letter; we will change the online submission form on your behalf. 3. Thank you for stating the following in your Competing Interests section: "None" Please complete your Competing Interests on the online submission form to state any Competing Interests. If you have no competing interests, please state ""The authors have declared that no competing interests exist."", as detailed online in our guide for authors at http://journals.plos.org/plosone/s/submit-now   This information should be included in your cover letter; we will change the online submission form on your behalf. 4. In the online submission form, you indicated that your data is available only on request from a third party. Please note that your Data Availability Statement is currently missing  contact details for the third party, such as an email address or a link to where data requests can be made. Please update your statement with the missing information.  5. Please amend the manuscript submission data (via Edit Submission) to include author Dr. Wei-Lin Lee. 6. Please include your full ethics statement in the ‘Methods’ section of your manuscript file. In your statement, please include the full name of the IRB or ethics committee who approved or waived your study, as well as whether or not you obtained informed written or verbal consent. If consent was waived for your study, please include this information in your statement as well. 7. Please include your tables as part of your main manuscript and remove the individual files. Please note that supplementary tables (should remain/ be uploaded) as separate ""supporting information"" files 8. We note you have included a table to which you do not refer in the text of your manuscript. Please ensure that you refer to Table 1 in your text; if accepted, production will need this reference to link the reader to the Table. 9. Please include captions for your Supporting Information files at the end of your manuscript, and update any in-text citations to match accordingly. Please see our Supporting Information guidelines for more information: http://journals.plos.org/plosone/s/supporting-information. 10. Please review your reference list to ensure that it is complete and correct. If you have cited papers that have been retracted, please include the rationale for doing so in the manuscript text, or remove these references and replace them with relevant current references. Any changes to the reference list should be mentioned in the rebuttal letter that accompanies your revised manuscript. If you need to cite a retracted article, indicate the article’s retracted status in the References list and also include a citation and full reference for the retraction notice.

[Note: HTML markup is below. Please do not edit.]

Reviewers' comments:

Reviewer's Responses to Questions

**Comments to the Author**

1. Is the manuscript technically sound, and do the data support the conclusions?

The manuscript must describe a technically sound piece of scientific research with data that supports the conclusions. Experiments must have been conducted rigorously, with appropriate controls, replication, and sample sizes. The conclusions must be drawn appropriately based on the data presented. 

Reviewer #1: Yes

Reviewer #2: Partly

********** 

2. Has the statistical analysis been performed appropriately and rigorously? 

Reviewer #1: Yes

Reviewer #2: Yes

********** 

3. Have the authors made all data underlying the findings in their manuscript fully available?

The PLOS Data policy requires authors to make all data underlying the findings described in their manuscript fully available without restriction, with rare exception (please refer to the Data Availability Statement in the manuscript PDF file). The data should be provided as part of the manuscript or its supporting information, or deposited to a public repository. For example, in addition to summary statistics, the data points behind means, medians and variance measures should be available. If there are restrictions on publicly sharing data—e.g. participant privacy or use of data from a third party—those must be specified.

Reviewer #1: No

Reviewer #2: Yes

********** 

4. Is the manuscript presented in an intelligible fashion and written in standard English?

PLOS ONE does not copyedit accepted manuscripts, so the language in submitted articles must be clear, correct, and unambiguous. Any typographical or grammatical errors should be corrected at revision, so please note any specific errors here.

Reviewer #1: Yes

Reviewer #2: Yes

********** 

5. Review Comments to the Author

Please use the space provided to explain your answers to the questions above. You may also include additional comments for the author, including concerns about dual publication, research ethics, or publication ethics. (Please upload your review as an attachment if it exceeds 20,000 characters)

Reviewer #1: This well written study focused on people with autism receiving HUD housing assistance. By connecting medicaid claim data and HUD data, the authors were able to explore the topic in detail.

As this paper is concise, well written, and of descriptive nature, I only have minor comments:

1. Some readers might not be familiar with various housing assistance programs provided via HUD, a brief intro on those programs can be very helpful.

2. I am not familiar with both datasets used in the study. I was wondering are there cases, if so, how many, are. people within the same household receiving HUD support?

3 I appreciate that the authors' statement on racial minority individuals were over-represented in the HUD assistance system signaling higher needs among this population. However, some may argue that this finding may indicate access of housing assistance among racial minorities, which counter the housing prioritization and allocation disparities faced by racial minorities identified from community stakeholders and research. I was wondering how would the authors address this concerns.

4. I appreciate the preemptive efforts suggested by the authors. I was wondering if the authors could discuss further what such interventions would look like and if there are existing models that the authors would recommend?

Reviewer #2: This paper examines the utilization of Housing and Urban Development (HUD) housing support for individuals with autism. This paper used Medicaid data from 2008 and 2016 and identified autistic individuals to then link them to national HUD data. Variables included demographic characteristic, co-occurring conditions, and HUD program involvement. I was hoping to see more variables (e.g., other disabilities and/or psychological disorders) included into this paper as part of co-occurring conditions. Overall, this paper examines a very important topic (i.e., role of housing assistance for autistic individuals) and has merit. I think this is a good start to begin discussions about HUD assistance for autistic individuals, particularly those who are underserved (homeless individuals and people of color). This paper can contribute good information to the roles that Medicaid benefits and HUD-assistance play in the health and mental outcomes within the developmental trajectories of individuals with autism.

Details:

Abstract

Line 48—HUD should be spelled since it is first mention.

Line 51—HUD should not be spelled out here but rather in line 48.

Introduction

Although the authors do a good job in highlighting the health and mental health outcomes (lines 77-78) within the autistic population, it would be helpful to add a few sentences pertaining specifically to mental and health outcomes (e.g., discrimination and racism) among marginalized individuals and/or people of color with autism (see Malone et al. 2022).

Can the authors include a few sentences to include autism spectrum symptoms (e.g., deficits in social communication and social interaction across multiple contexts)?

Can the authors include a few sentences that includes HUD eligibility? Eligibility may vary across states, but it would be important to include general eligibility criteria (e.g., low-income disabilities, U.S. citizens or eligible immigration status).

Page 2 first paragraph (line 68)—Can the authors provide citations for the “segregated datasets” they are alluding to?

Materials and Methods

Can the authors briefly clarify who developed the algorithm and how it developed? If necessary, please cite frameworks and/or previous studies from the research literature that contributed to the development of the algorithm.

Can the authors clarify in this section how they define co-occurring conditions?

Results

Page 4 second paragraph (line 125-126). If these data are available, can the authors include data pertaining to HUD-assisted individuals from other ethnoracial populations (e.g. Hispanic/Latinos, Native Americans, Asians)?

Discussion

Can the authors elaborate on the finding that 72.2% of HUD-assisted autistic were children under age 18. Why do the authors think this was the case and what are the practical implications of this?

Page 5 paragraph 2 (lines 145-145). Can the authors add statistics pertaining to homeless autistic individuals in the U.S.?

Page 5 paragraph 2 (lines 152-153). “…including increased exposure to environmental and social stressors that exacerbate health disparities.” Please add citations.

********** 

6. PLOS authors have the option to publish the peer review history of their article (what does this mean?). If published, this will include your full peer review and any attached files.

If you choose “no”, your identity will remain anonymous but your review may still be made public.

**Do you want your identity to be public for this peer review?** For information about this choice, including consent withdrawal, please see our Privacy Policy.

Reviewer #1: No

Reviewer #2: No

**********

[NOTE: If reviewer comments were submitted as an attachment file, they will be attached to this email and accessible via the submission site. Please log into your account, locate the manuscript record, and check for the action link "View Attachments". If this link does not appear, there are no attachment files.]

While revising your submission, please upload your figure files to the Preflight Analysis and Conversion Engine (PACE) digital diagnostic tool, https://pacev2.apexcovantage.com/. PACE helps ensure that figures meet PLOS requirements. To use PACE, you must first register as a user. Registration is free. Then, login and navigate to the UPLOAD tab, where you will find detailed instructions on how to use the tool. If you encounter any issues or have any questions when using PACE, please email PLOS at figures@plos.org. Please note that Supporting Information files do not need this step.

# Author comment: Author response to Decision Letter 0

**DOI:** 10.1371/journal.pone.0315008.r002

7 Nov 2024

November 6, 2024

Dear Dr. Chiyaka and reviewers, 

Thank you to Plos One for considering publication of our manuscript “Colliding Public Health Priorities: A Call to Improve the Understanding of Autistic Individuals Utilizing Housing Assistance" (PONE-D024-33807).” We are thrilled to hear of the overall positive review and potential publication. 

Please find the revised manuscript attached with changes detailed in the table below. We feel that the thorough feedback from the reviewers helped us to improve the overall quality of this study and the manuscript. 

Responses to Reviewer Feedback (RA-19-03-544)

Editor

Topic/Section Reviewer comment Response Action Taken

Keywords It seems that your current keywords, such as "disability" and "policy," do not align with the content of your article, as these terms are not mentioned anywhere in the text. Remember, your keywords should accurately reflect the central themes of your research and make your article more discoverable online. Consider revising your keywords to better describe the scope and findings of your study, ensuring they help people find your work when conducting relevant searches.

 Agreed Revised key words to: autism, housing, Medicaid

Role of Funder Please state what role the funders took in the study. If the funders had no role, please state: ""The funders had no role in study design, data collection and analysis, decision to publish, or preparation of the manuscript."" 

If this statement is not correct you must amend it as needed. 

Please include this amended Role of Funder statement in your cover letter; we will change the online submission form on your behalf.

 Updated We have added the following statement to our acknowledgements: “The funders had no role in study design, data collection and analysis, decision to publish, or preparation of the manuscript."

Competing Interests Add a competing interests statement Agreed Added: "The authors have declared that no competing interests exist."

Data Availability Statement Please note that your Data Availability Statement is currently missing contact details for the third party, such as an email address or a link to where data requests can be made. Completed We have added detail and the web link to the data center that manages CMS data requests. 

Authors Amend the manuscript submission data (via Edit Submission) to include author Dr. Wei-Lin Lee Completed Wei-Lin Lee has been added to the author list in the manuscript submission data. 

Ethics Statement In your statement, please include the full name of the IRB or ethics committee who approved or waived your study, as well as whether or not you obtained informed written or verbal consent. If consent was waived for your study, please include this information in your statement as well Completed Added to the Methods as requested

Tables/Files Please include your tables as part of your main manuscript and remove the individual files. Please note that supplementary tables (should remain/ be uploaded) as separate ""supporting information"" files.

Please ensure that you refer to Table 1 in your text Completed Added reference to Table 1 in the Results section and placed Table 1 after the first paragraph in which it is mentioned. 

Captions Please include captions for your Supporting Information files at the end of your manuscript, and update any in-text citations to match accordingly. We have added information to the end of the manuscript. 

Citations Please review your reference list to ensure that it is complete and correct. If you have cited papers that have been retracted, please include the rationale for doing so in the manuscript text, or remove these references and replace them with relevant current references. Any changes to the reference list should be mentioned in the rebuttal letter that accompanies your revised manuscript. Completed Completed

Reviewer 1

Reviewer #1: This well written study focused on people with autism receiving HUD housing assistance. By connecting medicaid claim data and HUD data, the authors were able to explore the topic in detail.

As this paper is concise, well written, and of descriptive nature, I only have minor comments.

Topic/Section Reviewer comment Response Action Taken

Introduction Some readers might not be familiar with various housing assistance programs provided via HUD, a brief intro on those programs can be very helpful. Agreed We have added text about HUD programs to the introduction. 

Introduction I am not familiar with both datasets used in the study. I was wondering are there cases, if so, how many, are. people within the same household receiving HUD support?

 Agreed We have added the number of individuals in the same household to the Results section. 

Discussion? I appreciate that the authors' statement on racial minority individuals were over-represented in the HUD assistance system signaling higher needs among this population. However, some may argue that this finding may indicate access of housing assistance among racial minorities, which counter the housing prioritization and allocation disparities faced by racial minorities identified from community stakeholders and research. I was wondering how would the authors address this concerns.

 This is an important comment and consideration. We sought in our text to denote increased exposure to disadvantages among racial minority individuals. We agree that access is likely a key consideration. We have added text to the statement in the discussion to underscore that barriers to service access is an additional potential factor exacerbating health inequities. 

Discussion I appreciate the preemptive efforts suggested by the authors. I was wondering if the authors could discuss further what such interventions would look like and if there are existing models that the authors would recommend?

 There is a limited evidence base to build upon for intervention opportunities. We believe that an existing gap in the field of autism research and practice that should including housing considerations and does not adequately do so currently includes assessment and service planning tools. We have added text to address. 

Reviewer 2

Reviewer #2: This paper examines the utilization of Housing and Urban Development (HUD) housing support for individuals with autism. This paper used Medicaid data from 2008 and 2016 and identified autistic individuals to then link them to national HUD data. Variables included demographic characteristic, co-occurring conditions, and HUD program involvement. I was hoping to see more variables (e.g., other disabilities and/or psychological disorders) included into this paper as part of co-occurring conditions. Overall, this paper examines a very important topic (i.e., role of housing assistance for autistic individuals) and has merit. I think this is a good start to begin discussions about HUD assistance for autistic individuals, particularly those who are underserved (homeless individuals and people of color). This paper can contribute good information to the roles that Medicaid benefits and HUD-assistance play in the health and mental outcomes within the developmental trajectories of individuals with autism.

Topic/Section Reviewer comment Response Action Taken

Abstract Spell out HUD name in first mention

 Agree Corrected

Introduction Although the authors do a good job in highlighting the health and mental health outcomes (lines 77-78) within the autistic population, it would be helpful to add a few sentences pertaining specifically to mental and health outcomes (e.g., discrimination and racism) among marginalized individuals and/or people of color with autism (see Malone et al. 2022).

 Agree We have added text to denote the presence and outline characteristics of these outcomes among individuals and people of color with autism. 

Introduction Can the authors include a few sentences to include autism spectrum symptoms (e.g., deficits in social communication and social interaction across multiple contexts)?

 Agreed We have added text to indicate the symptoms in from the reviewer comment. 

Introduction Can the authors include a few sentences that includes HUD eligibility? Eligibility may vary across states, but it would be important to include general eligibility criteria (e.g., low-income disabilities, U.S. citizens or eligible immigration status). Agreed We have added text to describe HUD eligibility criteria, which does not vary by state since HUD is federally administered. 

Introduction Page 2 first paragraph (line 68)—Can the authors provide citations for the “segregated datasets” they are alluding to? Agreed We have modified text to detail that data reflect the siloed systems from which they are generated. 

Methods Can the authors briefly clarify who developed the algorithm and how it developed? If necessary, please cite frameworks and/or previous studies from the research literature that contributed to the development of the algorithm.

 Agreed We have added text to indicate the location and ownership of the algorithms, including a cited link to the source for user documentation. 

Methods Can the authors clarify in this section how they define co-occurring conditions?

Agreed We have specified the source of the algorithm. 

Results Page 4 second paragraph (line 125-126). If these data are available, can the authors include data pertaining to HUD-assisted individuals from other ethnoracial populations (e.g. Hispanic/Latinos, Native Americans, Asians)?

 Agreed We have added a reference to Table 1 in this section for the full distribution of ethnoracial populations. 

Discussion Can the authors elaborate on the finding that 72.2% of HUD-assisted autistic were children under age 18. Why do the authors think this was the case and what are the practical implications of this?

 Thank you for this question. The disproportionate rate of children may point to lack of capacity to serve autistic adults, as seen in other systems. We added text and a citation to address this point. 

Discussion Page 5 paragraph 2 (lines 145-145). Can the authors add statistics pertaining to homeless autistic individuals in the U.S.?

 n/a There are no reliable published statistics on this population.

Discussion Page 5 paragraph 2 (lines 152-153). “…including increased exposure to environmental and social stressors that exacerbate health disparities.” Please add citations.

 Addressed Added a citation for this fact.

# Editor report: Decision Letter 1

**DOI:** 10.1371/journal.pone.0315008.r003

**Authors:** Edward Chiyaka

20 Nov 2024

Colliding Public Health Priorities: A Call to Improve the Understanding of Autistic Individuals Utilizing Housing Assistance

PONE-D-24-33807R1

Dear Dr. Shea,

We’re pleased to inform you that your manuscript has been judged scientifically suitable for publication and will be formally accepted for publication once it meets all outstanding technical requirements.

Within one week, you’ll receive an e-mail detailing the required amendments. When these have been addressed, you’ll receive a formal acceptance letter and your manuscript will be scheduled for publication.

An invoice will be generated when your article is formally accepted. Please note, if your institution has a publishing partnership with PLOS and your article meets the relevant criteria, all or part of your publication costs will be covered. Please make sure your user information is up-to-date by logging into Editorial Manager at Editorial Manager® and clicking the ‘Update My Information' link at the top of the page. If you have any questions relating to publication charges, please contact our Author Billing department directly at authorbilling@plos.org.

If your institution or institutions have a press office, please notify them about your upcoming paper to help maximize its impact. If they’ll be preparing press materials, please inform our press team as soon as possible -- no later than 48 hours after receiving the formal acceptance. Your manuscript will remain under strict press embargo until 2 pm Eastern Time on the date of publication. For more information, please contact onepress@plos.org.

Kind regards,

Edward Chiyaka, Ph.D., MSc

Academic Editor

PLOS ONE

Additional Editor Comments (optional):

Reviewers' comments:

# Editor report: Acceptance letter

**DOI:** 10.1371/journal.pone.0315008.r004

**Authors:** Edward Chiyaka

9 Dec 2024

PONE-D-24-33807R1 

PLOS ONE

Dear Dr. Shea, 

I'm pleased to inform you that your manuscript has been deemed suitable for publication in PLOS ONE. Congratulations! Your manuscript is now being handed over to our production team.

At this stage, our production department will prepare your paper for publication. This includes ensuring the following:

* All references, tables, and figures are properly cited

* All relevant supporting information is included in the manuscript submission,

* There are no issues that prevent the paper from being properly typeset

If revisions are needed, the production department will contact you directly to resolve them. If no revisions are needed, you will receive an email when the publication date has been set. At this time, we do not offer pre-publication proofs to authors during production of the accepted work. Please keep in mind that we are working through a large volume of accepted articles, so please give us a few weeks to review your paper and let you know the next and final steps. 

Lastly, if your institution or institutions have a press office, please let them know about your upcoming paper now to help maximize its impact. If they'll be preparing press materials, please inform our press team within the next 48 hours. Your manuscript will remain under strict press embargo until 2 pm Eastern Time on the date of publication. For more information, please contact onepress@plos.org.

If we can help with anything else, please email us at customercare@plos.org.

Thank you for submitting your work to PLOS ONE and supporting open access. 

Kind regards, 

PLOS ONE Editorial Office Staff

on behalf of

Dr. Edward Chiyaka 

Academic Editor

PLOS ONE
